# Hemp (*Cannabis sativa* L.) Protein Hydrolysates Promote Anti-Inflammatory Response in Primary Human Monocytes

**DOI:** 10.3390/biom10050803

**Published:** 2020-05-22

**Authors:** Noelia M. Rodriguez-Martin, Sergio Montserrat-de la Paz, Rocio Toscano, Elena Grao-Cruces, Alvaro Villanueva, Justo Pedroche, Francisco Millan, Maria C Millan-Linares

**Affiliations:** 1 Department of Medical Biochemistry, Molecular Biology, and Immunology, School of Medicine, Universidad de Sevilla, 41009 Seville, Spain; noe91rm@gmail.com (N.M.R.-M.); delapaz@us.es (S.M.-d.l.P.); mtoscanos@us.es (R.T.); elenagraoc@gmail.com (E.G.-C.); 2 Department of Food & Health, Instituto de la Grasa, CSIC, 41013 Seville, Spain; alvarovillanueva@ig.csic.es (A.V.); jjavier@cica.es (J.P.); fmillanr@ig.csic.es (F.M.); 3 Cell Biology Unit, Instituto de la Grasa, CSIC, 41013 Seville, Spain

**Keywords:** hemp seed, protein hydrolysates, peptides, inflammation, monocytes, polarization, microglia

## Abstract

Hemp seeds have a wide variety of chemical compounds which present biological activity. Specifically, the focus on proteins and bioactive peptides are increasing as alternative sources of nutraceutical uses. In the literature, hemp protein products (HPPs) have reported antioxidant and anti-inflammatory properties. This study aimed to determine the inflammation-related modulatory effects of HPPs on lipopolysaccharide (LPS)-activated primary human monocytes. CD14^+^ cells were immunomagnetically isolated from buffy coats and the anti-inflammatory activity of hemp protein isolate (HPI) and hydrolysates (HPHs) was evaluated on LPS-stimulated human primary monocytes. The specific markers of inflammation, polarization, and chemoattraction were measured by RT-qPCR and ELISA assays. Our results showed that HPPs decreased the pro-inflammatory mediators (*TNF-α*, *IL-1β*, and *IL-6*) and increased the anti-inflammatory mediators (*IL-10* and *IL-4*). In addition, M1 polarization marker gene expression (*CCR7* and *iNOS*) was downregulated by HPPs and, M2 polarization marker gene expression (*CD200R* and *MRC1*) was upregulated. Finally, the mRNA expression of chemotaxis genes (*CCR2* and *CCL2*) was downregulated by HPPs. In conclusion, this study suggests that HPPs may improve chronic inflammatory states and promote regenerative processes by reprogramming monocytes toward M2 polarization phenotype.

## 1. Introduction

*Cannabis sativa* L. has been widely grown for several millennia [1]. Industrially, the plant is used for a stem fiber (also called hemp) and an edible oil derived from seeds. Plant varieties utilized for fiber and oilseed usually are low in the euphoria-inducing chemical named Δ9-tetrahydrocannabinol (THC) compared to drug varieties [2,3]. Hemp crop is legal since 1993 in many European countries. The cultivation area is even expanding, from 8000 ha in 2011 to reach more than 33,000 ha in 2016. The main reason for this crop to be enhanced is the environmental benefits; hemp is a valuable crop for less work time, lower costs, and greater profit, which improve the bio-based economy [4]. In addition, the Grand View Research (2020) reported that the economy of hemp crop will be increased, with at least USD 15 billion in 2027 [5]., due to hemp being the source of 25,000 biodegradable products which have increasing uses [4]. In recent years, hemp crop is increasing not only for industrial uses, but also for its health benefits. Due to multiple phytochemical compounds hemp seeds have also reached a greater degree of international scientific attention [6,7,8]. In particular, protein represents around 25% of the total hemp seed composition [9]. The major fraction of the total seed protein consists of storage proteins as globulins (65–75%), albumins (25–37%), and sulfur-rich proteins [10]. The most significant protein in hemp seed is edestin, a globulin rich in arginine and a recognized precursor of nitric oxide, which makes hemp seed protein of high interest to enhance cardiovascular health [10]. Nevertheless, the poor water solubility of hemp proteins are an obstacle for their use, which was technologically overwhelmed by enzymatic hydrolysis [11]. Hemp protein hydrolysates (HPHs) generated by enzymatic hydrolysis are composed of polypeptides, oligopeptides, and free amino acids displaying high availability of biological activity [8]. In the literature, HPHs have showed different biological activities as antihypertensive, hypocholesterolemic, antioxidant, antithrombotic, and immunomodulatory effects [8,9,11,12,13,14,15]. In addition, neuroprotective HPHs have been recently described in preliminary studies by our research team in a microglial BV2 cell line stimulated with lipopolysaccharide (LPS) [9]. However, HPHs’ effects have not yet been investigated in primary human blood circulating cells.

Peripheral blood mononuclear cells (PBMCs) are particularly linked to systemic inflammation and several diseases [16,17,18,19]. PBMCs include lymphoid cells as lymphocytes (T cells, B cells, and NK cells) and myeloid cells as monocytes. Monocytes are mononuclear cells that develop in the bone marrow and diffuse inside of the bloodstream. Monocytes have a plastic nature, which are capable of a notable range of phenotypic and functional changes which are conditioned by signals from local microenvironments [20,21]. In humans, monocytes are classified into three defined subsets: classical monocytes (CD14^++^CD16^−^), intermediate monocytes (CD14^++^CD16^+^), and non-classical monocytes (CD14^+^CD16^++^) [22]. As described previously, the polarization of macrophage-like cells from monocyte is classified into classically (M1) and alternatively (M2) activated groups [23]. Classical monocytes-derived M1 macrophages provoke the production of inflammatory cytokines and reactive nitrogen or oxygen reactive species that contribute to host defense, whereas non-classical monocytes-derived M2 macrophages attenuate inflammation and participate in tissue renovation. Regarding the current research, we further investigated whether hemp protein products (HPPs) may act as an immune-modulator and anti-inflammatory effector on human primary monocytes.

## 2. Materials and Methods

### 2.1. Chemical Compounds and Sampling

Seeds of *Cannabis sativa* L. were purchased from Sensi Seeds Bank. Alcalase 2.4 L (2.4 AU/g) and flavourzyme (1000 L) were a gift from Novozymes (Bagsvaerd, Denmark). Ficoll Histopaque and LPS (*E. coli* 055: B5) were provided by Sigma-Aldrich Chemistry. TRIsure (Bioline, Meridian Life Science, Inc. Memphis, TN, USA) and cDNA synthesis kits from Bio-rad (Berkeley, CA, USA) were used for RNA isolation. Quantitative PCR was performed by iTaq™ Universal SYBR^®^ Green Supermix from Bio-rad. Primers were provided by Eurofins Biolab S.L.U (Barcelona, Spain). All reagents and solvents were of analytical grade and provided by Sigma Chemical Co. (St. Louis, MO, USA), Bachem AG (Bubendorf, Switzerland), and Gibco (Paisley, UK).

### 2.2. Hemp Protein Products Preparation

HPPs, that include hemp protein isolate (HPI) and hydrolysates (HPHs), were extendedly explained in preliminary studies as well as their chemical characterization [9]. Briefly, to obtain the HPI, the first step was defatting with n-hexane in a soxhlet, then was performed a first solubilization in alkali at pH 10.5 for 1 h, and, finally, the protein isolate was obtained by precipitation at the isoelectric point (pH 4.3). Second, the HPI was treated with proteases as alcalase or llavourzyme to obtain the different HPHs in a bioreactor under controlled conditions. In line with our previous results, HPI and HPHs with better ex-vivo antioxidant properties and in-vitro anti-neuroinflammatory potential, HPH20A and HPH60A15F, were used in this study. The HPH20A was obtained from HPI hydrolyzed during 20 min with alcalase; and HPH60A15F, was obtained from HPI hydrolyzed during 60 min with alcalase and an additional 15 min with alcalase+flavourzyme. These hydrolysates were boiled at 85 °C for 15 min to stop the enzymatic reaction. Finally, the supernatant was centrifugated (7500 rpm, 15 min) to obtain the HPHs. The resulting hydrolysates were composed of a mixture of amino acids and small peptides with biological activity. As an example, the HPHs were rich in negatively charged amino acids like asparagine and aspartic acid, and glutamine and glutamic acid, and also rich in arginine [9]. The chemical composition and amino acid characterization of hemp seed and HPPs are shown in the Appendix A (Table A1 and Table A2).

### 2.3. Blood Collection and Monocyte Isolation

To carry out this study, we used the Good Clinical Practice Guidelines procedure with the principles outlined in the Helsinki Declaration of the World Medical Association [24]. Peripheral blood mononuclear blood cells (PBMCs) were centrifugated over a Ficoll–Histopaque gradient isolated from buffy coat. Blood was donated by the Regional Blood Transfusion Center (Seville, Spain. Agreement #33130099). The donors were male, 25–35 years, declared themselves as non-smokers, and they were not taking any medication. Monocytes were isolated from PBMCs using CD14 MicroBeads (MACS, Myltenyi). Flow cytometry (FACScanto II flow cytometer and FACSDiva software, BD) showed a purity of the CD14 monocytes isolations > 95%, routinely. Following isolation, monocytes were maintained at Roswell Park Memorial Institute (RPMI) in 1640 medium enriched with L-glutamine, 10% heat-inactivated foetal bovine serum, and 1% penicillin/streptomycin in 5% CO_2_ at 37 °C in a CO_2_ incubator (Thermo Con Electron Corporation, Waltham, MA, USA).

### 2.4. Treatments of Cells in Culture

After isolation, monocytes (5 × 10^5^ cells/well) were incubated with LPS (100 ng/mL) followed or not by 24 h treatment in 24-well plates with HPI, HPH20A, and HPH60A15F at 50 and 100 µg/mL. At the end of exposure time, supernatants were extracted and stored at −20 °C, and RNA was obtained using these cells.

### 2.5. Measurement of Cytokine Release

The supernatant was obtained in order to quantify the cytokine levels of *TNF-α*, *IL-1β*, *IL-6*, and *IL-10* by the enzyme-linked immunosorbent assay (ELISA), using the instructions of the manufacturer protocol (Diaclone, Besancon, France). The amount of cytokine was calculated from calibration standard curves and expressed in pg per mL.

### 2.6. RNA Isolation and RT-qPCR Analysis

RNA from monocytes was isolated to quantify gene expression by RT-qPCR. Total RNA was obtained by using TRIsure Reagent. RNA quality was evaluated by A_260_/A_280_ ratio in a NanoDrop ND-1000 Spectrophotometer (ThermoFisher Scientific, Madrid, Spain). After that, the RNA was subjected to reverse transcription. The resulting cDNA was used in an amount of 10 g for RT-qPCR amplifications. A CFX96 system (Bio-Rad) was used to determine the mRNA levels for genes indicated below. For each PCR reaction, the Brilliant SYBR green QPCR Supermix containing the primer pairs for either gene, or for glyceraldehyde 3-phosphate dehydrogenase (*GAPDH*) and hypoxanthine phosphoribosyltransferase (*HPRT*) as housekeeping genes, was added to cDNA template. All PCR amplifications were accomplished in three identical copies and average threshold cycle (Ct) numbers of the triplicates were used to determine the relative mRNA expression of selected genes. The magnitude of change of mRNA expression for selected genes was assessed by using the 2^−(ΔΔCt)^ mathematical method. All data were normalized to housekeeping gene constituents and expressed as relative fold-change of control. The designed oligonucleotides sequences are shown in Table 1.

### 2.7. Statistical Method

All values are expressed as arithmetic means ± standard deviations (SD). The output values were evaluated with Graph Pad Prism Version 5.01 software (San Diego, CA, USA). The one-way analysis of variance (ANOVA) was used to evaluate statistical significance of any difference in each parameter among the groups, following Tukey multiple comparisons test as *post hoc* tests. *p* values considered statistically significant were less than 0.05.

## 3. Results

### 3.1. Anti-Inflammatory Properties of Hemp Protein Hydrolysates

**Figure 1:**
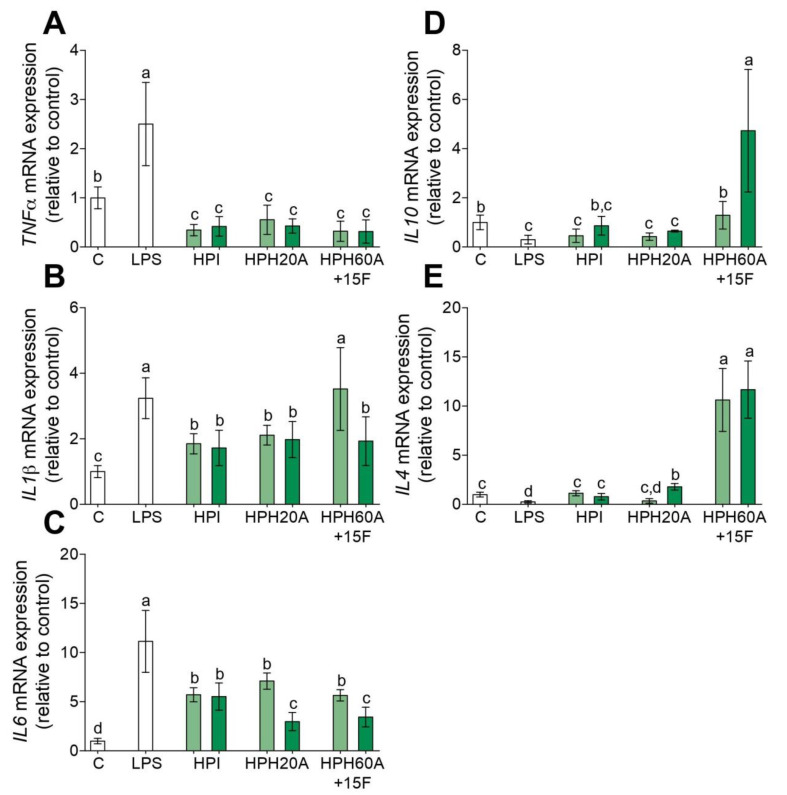
Effect of hemp protein products (HPPs) on inflammatory cytokine gene expression in LPS-stimulated primary human monocytes. Monocytes were treated with LPS (100 ng/mL) and then incubated with hemp protein isolate (HPI), HPH20A, and HPH60A15F at 50 (light green bar) and 100 (dark green bar) μg/mL for 24 h. Relative mRNA expression levels of *TNF-α* (**A**), *IL-1β* (**B**), *IL-6* (**C**), *IL-10* (**D**), and *IL-4* (**E**) were detected by real-time quantitative PCR. Data are show as means ± SD (*n* = 3) and the significantly different (*p* < 0.05) are labeled with contrasting letters.

**Figure 2:**
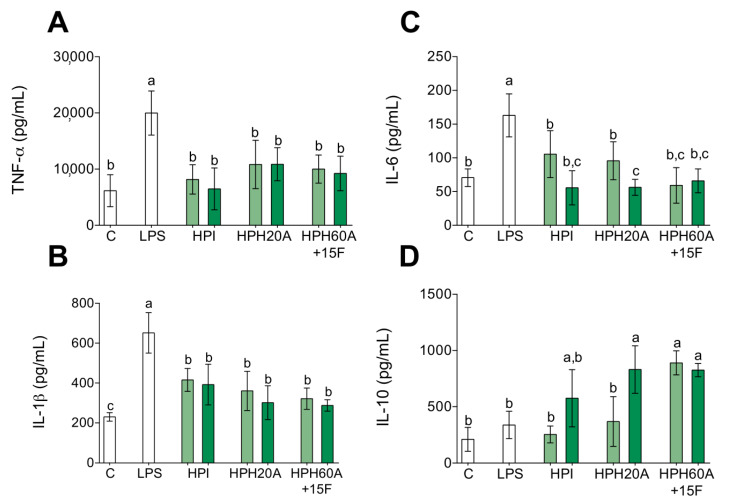
Effect of HPPs on inflammatory cytokine release in LPS-stimulated primary human monocytes. Monocytes were activated with LPS (100 ng/mL) and then treated with HPI, HPH20A, and HPH60A15F at 50 (light green bar) and 100 (dark green bar) μg/mL for 24 h. Concentration of *TNF-α* (**A**), *IL-1β* (**B**), *IL-6* (**C**), and *IL-10* (**D**) in culture supernatants was detected by ELISA. Data are presented as means ± SD (*n* = 3) and the significantly different (*p* < 0.05) are labeled with contrasting letters.

Chronic inflammation is key in several disorders [16,17,18,25,26,27,28,29,30,31] and the activation of circulating monocytes is a recognized process during inflammation [18,19]. Therefore, to assess the anti-inflammatory effect of HPHs, gene expression of *TNF-α*, *IL-1β*, *IL-6*, *IL-10*, and *IL-4* was measured by RT-qPCR in LPS-stimulated primary human monocytes. As shown in Figure 1A–C, the inflammation induced by LPS upregulates the mRNA levels of *TNF-α*, *IL-1β*, and *IL-6* compared to those in untreated cells. However, this inflammatory state is blocked with the presence of HPI, HPH20A, and HPH60A15F at 50 or 100 µg/mL. In addition, the mRNA levels of the anti-inflammatory cytokines *IL-10* (Figure 1D) and *IL-4* (Figure 1E) are upregulated with the presence of HPH60A15F at 50 or 100 µg/mL. However, the treatment with HPI and HPH20A does not show an upregulation of these anti-inflammatory genes compared to HPH60A15F. At higher concentration, HPI slightly upregulates *IL-10*, meanwhile HPI and HPH20A slightly upregulate *IL4* in LPS-stimulated primary human monocytes. This capacity of HPPs to decrease the transcriptional activity of pro-inflammatory genes and to increase the transcriptional activity of anti-inflammatory genes, is also linked with a reduced release of *TNF-α* (Figure 2A), *IL-1β* (Figure 2B), and *IL-6* (Figure 2C) cytokines and an increased release of *IL-10* (Figure 2D) into cell culture supernatant.

### 3.2. Hemp Protein Hydrolysates Shifts M1 toward M2 Macrophage Phenotype

**Figure 3:**
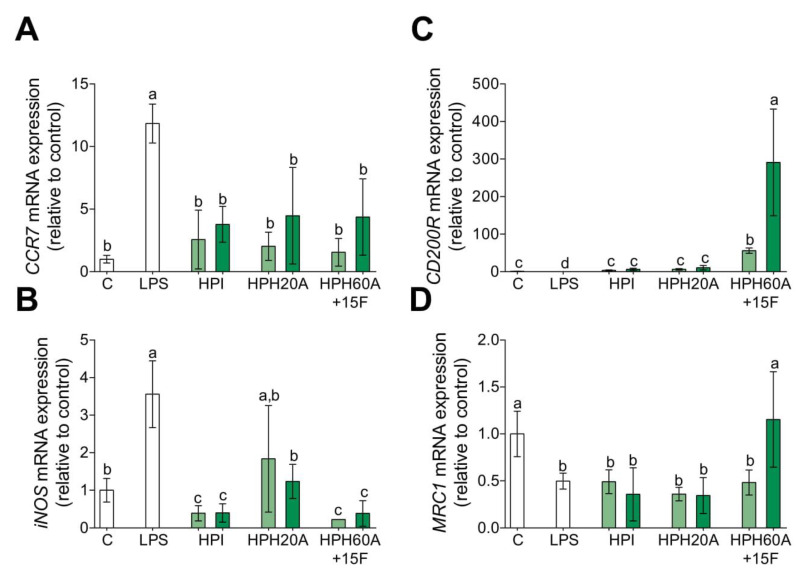
Effect of HPPs on macrophage polarization marker gene expression in LPS-stimulated primary human monocytes. Monocytes were activated with LPS (100 ng/mL) and then treated with HPI, HPH20A, and HPH60A15F at 50 (light green bar) and 100 (dark green bar) μg/mL for 24 h. Relative mRNA expression levels of M1 markers, *CCR7* (**A**) and *iNOS* (**B**), and M2 markers, *CD200R* (**C**) and *MRC1* (**D**), were detected by real-time quantitative PCR. Data are presented as means ± SD (*n* = 3) and the significantly different (*p* < 0.05) are labeled with contrasting letters.

The cytokine microenvironment of monocytes may define the polarization state toward M1 or M2 macrophage phenotypes. The cytokines associated to M1 phenotype are pro-inflammatory cytokines such as *TNF-α*, *IL-1β*, and *IL-6*. Hence, the current classification between M1 and M2 macrophages gives emphasis to the expression of other genes which are related to their functional properties [21,23]. Gene expression of phenotypic markers for M1, such as *CCR7* and *iNOS*, and phenotypic markers for M2, such as *CD200R* and *MRC1*, was investigated. As shown in Figure 3A,B, the transcriptional mRNA levels of *CCR7* and *iNOS* are upregulated with LPS. However, HPHs downregulate the transcriptional mRNA levels of M1 polarization marker genes. In contrast, HPHs particularly promote *CD200R* (Figure 3C) and *MRC1* (Figure 3D) transcriptional activity. These results may indicate that HPPs, particularly HPH60A15F, shift macrophage polarization from M1 toward M2.

### 3.3. Hemp Protein Hydrolysates Modulates CCR2-Dependent Migration

**Figure 4:**
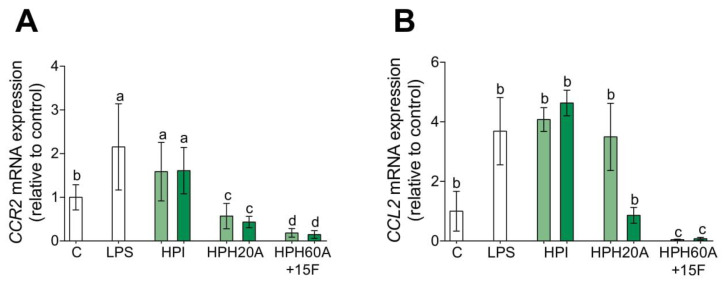
Effect of HPPs on chemotactic CCR2/CCL2 gene expression in LPS-stimulated primary human monocytes. Monocytes were activated with LPS (100 ng/mL) and then treated with HPI, HPH20A, and HPH60A15F at 50 (light green bar) and 100 (dark green bar) μg/mL for 24 h. Relative mRNA expression levels of *CCR2* (**A**) and *CCL2* (**B**) were detected by real-time quantitative PCR. Data are presented as means ± SD (*n* = 3) and the significantly different (*p* < 0.05) are labeled with contrasting letters.

In gaining deeper insight into the role of HPPs on human monocyte plasticity, we studied the modulation of the CCR2/CCL2 axis. Classically activated or M1 macrophages are recruited into the inflamed tissue via the *CCR2* signaling pathway, which regulates the monocyte migration. Conversely, the alternatively activated or M2 macrophages are characterized for their poor migratory ability [21,23]. In establishing the physiological relevance of this pathway, the mRNA expression of *CCR2* and *CCL2* genes in the presence of hemp protein products was analyzed by RT-qPCR (Figure 4). The mRNA levels of *CCR2* (Figure 4A) and *CCL2* (Figure 4B) are upregulated with LPS compared to those in untreated cells. The transcriptional activity of CCR2 and CCL2 are downregulated in the presence of HPHs but not in the presence of HPI. Notably, HPH60A15F significantly downregulates mRNA levels compared to HPH20A. These results might confirm that HPHs polarize primary human monocytes toward M2 poorly migratory anti-inflammatory macrophages.

## 4. Discussion

Current dietary habits are driven towards the consumption of ready-to-eat foods rich in refined sugars and unhealthy fats. Nutritional interventions have been consistently proposed as a part of a comprehensive strategy to lower the incidence and severity of several diseases. Excessive consumption of animal-based proteins is associated with an increased risk of atherosclerosis and cancer [29,30,31]. By contrast, replacement of animal- with plant-based proteins has been reported to be inversely associated with risk of atherosclerosis [30]. The industrial hempseed, i.e., the non-drug cultivars of *C. sativa*, is undoubtedly an underexploited protein-rich seed [32]. Hempseed proteins are an excellent natural source of digestible amino acids in comparison to other protein sources, such as borage meal, canola meal, and heated canola meal [33,34]. The literature evidence shows that the most effective HPPs identified so far are ACE-inhibitory peptides, which are composed of 2–20 amino acids. In addition, they should present good chemical properties such as the balance of hydrophobicity/hydrophilicity and some particular structural characteristics related to its sequence [13,32,35,36]. In previous results of our research team, we evaluated the potential antioxidant activity of the HPIs, HPHs with alcalase, and HPHs with alcalase and flavourzyme in cell-free in vitro and microglial BV2 experiments [9]. HPHs inhibited oxidative stress and inflammatory response in activated microglia, however, the immunomodulatory effects of HPHs have not yet been investigated in primary human monocytes.

Monocytes are determinative cells in the development and propagation of the inflammatory nature and atherosclerosis onset. The normal activity of primary human monocytes is known to be substantially disturbed by activation during the plaque formation [24,25]. Activation of monocytes is observed in chronic inflammatory processes, i.e., cardiovascular diseases. When monocytes are exposed to LPS, they produce several inflammatory mediators which are involved in different injuries and diseases, particularly in inflammation, metabolic, and vascular-related disorders [17,18,19]. During inflammation, the dual roles of monocytes are defined in part by two specific morphological phenotypes of macrophages that they have the tendency to be polarized into. The M1 macrophages stem from classical monocytes, which are rich in CD14 and show high expression of chemotaxis genes such as *CCR2* (C-C chemokine receptor 2) and *CCL2* (C-C ligand 2, also referred to as monocyte chemoattractant protein 1) [18,22]. On the other hand, the M2 macrophages stem from non-classical monocytes, which are rich in CD16 and show high expression of *CD200R* (Cell surface glycoprotein CD200 receptor) and *MRC1* (C-type mannose receptor 1) [21,23]. The M1 phenotype is a pro-inflammatory state and is involved in the production of inflammatory mediators, including pro-inflammatory cytokines (*IL-1β*, *TNF-α*, and *IL-6*) and chemokines, and induces oxidative stress damage by *iNOS* (inducible nitric oxide synthase) induction. Alternatively, the M2 phenotype is involved in the production of protective cytokines (*IL-4* and *IL-10*) and plays an important role in tissue repairs [21].

Given the importance of monocytes on innate immune function, our study highlights the effects of HPPs on the inflammatory response in LPS-activated primary human monocytes. In the literature, it is possible to find a representative number of cases where plant-derived biopeptides are used as anti-inflammatory or antioxidant chemicals. A model of them is 1,2,3,4,6 penta-O-galloyl-β-D-glucose, a naturally occurring polyphenolic chemical present in some therapeutical herbs as *Rhuschinensis Mill* [37]. One more example of a bioactive plant is *Fagopyrum tataricum*, widely named as buckwheat. Other isolated chemicals with anti-inflammatory activity in activated PBMCs (monocytes or macrophages) are the Brazilian red propolis (*Apis mellifera*), *Copaifera* oleoresins, *Citrus bergamia* juice flavonoid fraction, effusanin C (*Isodon japonicus*), and oligomeric proanthocyanidins (*Crataegus oxyacantha*) [38,39,40,41,42]. Lunasin, a 43-amino acid multifunctional bioactive peptide in soybean seeds, has been found to exert numerous biological activities, including anti-inflammatory and antioxidant properties, in human monocytic leukemia cell line THP-1 cells [43]. In addition, GPETAFLR, an octapeptide isolated from *Lupinus angustifolius* L., showed an anti-inflammatory effect in LPS-stimulated primary human monocytes [44]. In the present study HPHs have shown diminished inflammation in LPS-stimulated primary human monocytes. These observations are consistent with the mRNA levels of inflammatory cytokines, with additional contributions to the M2 phenotype polarization, as well as the CCR2-dependent migration markers. Hence, the activated macrophages treated with HPPs might target the inflammation and successfully prevent inflammatory states and inflammatory derived complications from several human diseases. This anti-inflammatory capacity may be a result of biopeptide presence or the synergistic association of free amino acids which exert primary antioxidant activity [8,9].

Our study is supplementary to a previous one in which HPPs were demonstrated to show an antioxidant ability in ex-vivo experiments as well as anti-inflammatory properties in murine BV-2 microglial cells [9]. However, the targeting pathways of the immune system by HPHs are unknown. Subsequent to these observations, the literature evidence have been suggested that the LPS-activated-macrophages promote pro-inflammatory pathways such as the nuclear factor (NF)-κB signaling pathway and the Janus-activated kinase (JAK)/STAT3-dependent pathway [17,29]. On this matter, some authors have observed that LPS is recognized by TLR4 (toll like receptor-4) in human monocytes [28,29]. This receptor could activate the NF-κB pathway. As a result, the activated pro-inflammatory pathways are involved in the release of *iNOS*. Our results showed that LPS-activated monocytes produce an increase in *iNOS* transcriptional levels, though the HPP treatments have induced a normalization of the *iNOS* transcriptional levels. In addition, other observations may drive the investigation to the nucleotide-binding domain and leucine-rich repeat containing protein (NLR) expression and the NLRP3 inflammasome activation pathway which regulate the secretion of *IL-1β* [20]. As was observed, *IL-1β* was increased in the LPS-activated monocytes, which may indicate NLRP3 inflammasome activation. At which time, further studies should be directed toward the targeting pro-inflammatory pathway in presence of HPHs.

## 5. Conclusions

**Figure 5:**
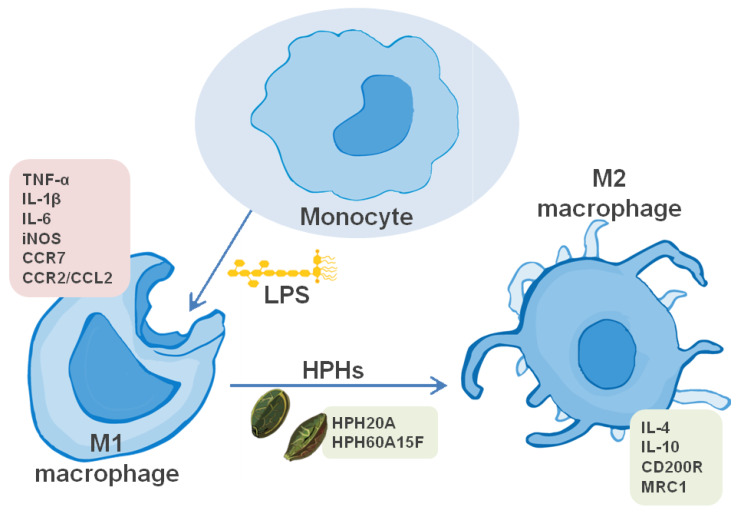
Schematic representation of the actions of HPHs in LPS-stimulated primary human monocytes. The scheme summarizes the hypothesis by which HPHs would acutely modulate the polarization of human monocyte into M2 macrophage. This model does not exclude the participation of additional mechanisms of action for HPHs to achieve their effects on the macrophage polarization.

Taken together, our outcomes suggest that both hydrolysates, HPH20A and HPH60A15F, have significant anti-inflammatory properties in LPS-activated primary human monocytes. Additionally, both hydrolysates have the capacity of regenerated inflammatory processes by an over-activation of M2 phagocytes, as is summarized in Figure 5. Therefore, the present study reveals new beneficial effects of hemp protein products, including HPH20A and HPH60A15F, as well as allows their potential uses in the prevention and treatment of inflammation-related conditions as natural bioactive compounds.

## Figures and Tables

**Table 1:** Primer sequences for RT-qPCR gene expression analysis.

Target	GeneBank Accession Number	Forward Reverse	Sequence (5′ → 3′)
*TNF-α*	NM_000594	Forward Reverse	TCCTTCAGACACCCTCAACC AGGCCCCAGTTTGAATTCTT
*IL-1β*	NM_000576	Forward Reverse	GGGCCTCAAGGAAAAGAATC TTCTGCTTGAGAGGTGCTGA
*IL-6*	NM_000600	Forward Reverse	TACCCCCAGGAGAAGATTCC TTTTCTGCCAGTGCCTCTTT
*IL-10*	NM_000572	Forward Reverse	GCCTAACATGCTTCGAGATC TGATGTCTGGGTCTTGGTTC
*IL-4*	NM_021283.2	Forward Reverse	TCAACCCCCAGCTAGTTGTC TGTTCTTCGTTGCTGTGAGG
*CCR7*	NM_007719.2	Forward Reverse	GTGTGCTTCTGCCAAGATGA CCACGAAGCAGATGACAGAA
*iNOS*	NM_ 000625	Forward Reverse	ACCCAGACTTACCCCTTTGG GCCTGGGGTCTAGGAGAGAC
*CD200R*	NM_138940.2	Forward Reverse	GTTGCCCTCCTATCGCATTA TGGAAATTCCCATCAGGTGT
*MRC1*	NM_ 138806	Forward Reverse	GGCGGTGACCTCACAAGTAT ACGAAGCCATTTGGTAAACG
*CCR2*	NM_001123396.1	Forward Reverse	TGCCTGACTCACACTCAAGG GGCTTCTCAGCAACTGAACC
*CCL2*	NM_002982.3	Forward Reverse	CCCCAGTCACCTGCTGTTAT ACGAAGCCATTTGGTAAACG
*HPRT*	NM_001289746	Forward Reverse	ACCCCACGAAGTGTTGGATA AAGCAGATGGCCACAGAACT
*GADPH*	NM_001289746	Forward Reverse	CACATGGCCTCCAAGGAGTAAG CCAGCAGTGAGGGTCTCTCT

## Appendix A

**Table A1:** Chemical composition of hemp seed, HPI, HPH20A and HPH60A15F. Data, expressed as percentage in dry basis, are mean ± standard deviation of triplicates [9].

Nutrient (g/100 g Product)	Hemp Seed	Hemp Protein Isolate (HPI)	Hemp Protein Hydrolysate: HPH20A	Hemp Protein Hydrolysate: HPH 60A + 15AF
Proteins	24.18 ± 1.01	96.46 ± 0.93	86.56 ± 0.78	83.02 ± 0.29
Carbs	3.81 ± 0.76	0.00 ± 0.00	0.00 ± 0.00	0.00 ± 0.00
Fat	32.35 ± 0.57	---------	---------	---------
Humidity	4.49 ± 0.01	2.19 ± 0.01	7.34 ± 0.03	8.85 ± 0.04
Ash	5.09 ± 0.07	1.06 ± 0.10	6.10 ± 0.75	8.13 ± 0.26
Fiber	29.72 ± 1.44	00.00 ± 0.00	0.00 ± 0.00	0.00 ± 0.00
Polifenols	0.35 ± 0.02	0.04 ± 0.00	0.00 ± 0.00	0.00 ± 0.00

**Table A2:** Amino acid profile of defatted hemp flour (DHF), HPI, HPH20A and HPH60A15F by HPLC. Values were expressed as a percentage of amino acids on the total amino acid content, are the mean ± standard deviation of triplicates [9].

Amino Acids (g/100 g Protein)	Defatted Hemp Flour (DHF)	Hemp Protein Isolate (HPI)	Hemp Protein Hydrolysate: HPH20A	Hemp Protein Hydrolysate: HPH 60A + 15AF
Aspartic acid + asparragine	11.34 ± 0.37	12.35 ± 0.34	12.40 ± 0.29	12.08 ± 0.51
Glutamic acid + Glutamine	18.41 ± 0.13	18.86 ± 0.35	18.83 ± 0.25	18.58 ± 0.12
Alanine	4.89 ± 0.10	5.01 ± 0.14	4.99 ± 0.10	5.00 ± 0.11
Arginine	12.68 ± 0.21	13.44 ± 0.03	13.34 ± 0.24	13.78 ± 0.23
Cysteine	1.22 ± 0.02	1.98 ± 0.08	1.81 ± 0.18	1.65 ± 0.21
Glycine	4.74 ± 0.07	4.79 ± 0.23	4.64 ± 0.11	4.64 ± 0.12
Histidine	3.07 ± 0.01	2.85 ± 0.05	2.80 ± 0.02	2.79 ± 0.03
Isoleucine	4.23 ± 0.08	3.02 ± 0.40	3.57 ± 0.15	3.85 ± 0.20
Leucine	7.12 ± 0.05	7.13 ± 0.11	7.12 ± 0.13	7.34 ± 0.07
Lysine	3.78 ± 0.09	3.04 ± 0.08	3.10 ± 0.06	3.23 ± 0.04
Methionine	2.14 ± 0.02	0.89 ± 0.34	0.60 ± 0.03	0.64 ± 0.04
Phenylalanine	4.76 ± 0.09	5.12 ± 0.02	5.04 ± 0.05	5.19 ± 0.08
Proline	2.66 ± 0.69	3.02 ± 0.00	2.83 ± 0.05	1.29 ± 0.19
Serine	5.57 ± 0.11	6.31 ± 0.21	6.36 ± 0.19	6.31 ± 0.22
Threonine	3.79 ± 0.08	3.79 ± 0.10	3.89 ± 0.06	4.02 ± 0.07
Tryptophan	0.76 ± 0.05	0.55 ± 0.02	0.66 ± 0.01	0.82 ± 0.03
Tyrosine	3.57 ± 0.06	4.08 ± 0.06	3.98 ± 0.03	4.06 ± 0.12
Valine	5.27 ± 0.11	3.77 ± 0.40	4.05 ± 0.42	4.71 ± 0.19
